# Phenotypic and functional characterization of switch memory B cells from patients with oligoarticular juvenile idiopathic arthritis

**DOI:** 10.1186/ar2824

**Published:** 2009-10-05

**Authors:** Anna Corcione, Francesca Ferlito, Marco Gattorno, Andrea Gregorio, Angela Pistorio, Roberto Gastaldi, Claudio Gambini, Alberto Martini, Elisabetta Traggiai, Vito Pistoia

**Affiliations:** 1 Laboratory of Oncology, IRCCS G. Gaslini, Largo G. Gaslini 5, Genoa, 16148, Italy; 2 Second Division of Pediatrics, IRCCS G. Gaslini, Largo G. Gaslini 5, Genoa, 16148, Italy; 3 University of Genoa, Viale Benedetto XV, Genoa, 16100, Italy; 4 Human Anatomy Section, IRCCS G. Gaslini, Largo G. Gaslini 5, Genoa, 16148, Italy; 5 Clinical Epidemiology and Biostatistics Unit, Scientific Direction, IRCCS G. Gaslini Institute, Largo G. Gaslini 5, Genoa, 16148, Italy; 6 IRCCS G. Gaslini, Largo G. Gaslini 5, Genoa, 16148, Italy

## Abstract

**Introduction:**

In chronic inflammatory disorders, B cells can contribute to tissue damage by autoantibody production and antigen presentation to T cells. Here, we have characterized synovial fluid and tissue B-cell subsets in patients with oligoarticular juvenile idiopathic arthritis (JIA), an issue not addressed before in detail.

**Methods:**

B cells from synovial fluid (SF) and peripheral blood (PB) of 25 JIA patients, as well as from PB of 20 controls of comparable age, were characterized by multicolor flow cytometry. Immunoglobulin-secreting cells were detected by ELISPOT. Immunohistochemical analyses of synovial tissue from three JIA patients were performed.

**Results:**

JIA SF B cells were enriched in CD27^+ ^and CD27^- ^switch memory B cells, but not in CD27^+ ^IgM memory B cells, compared with patient and control PB. Plasma blasts were more abundant in SF and secreted higher amounts of IgG. Lymphoid aggregates not organized in follicle-like structures were detected in synovial tissue sections and were surrounded by CD138^+ ^plasma cells. Finally, transitional B cells were significantly increased in JIA PB versus SF or control PB. CCR5, CCR8, CXCR2, and CXCR3 were upregulated, whereas CCR6, CCR7, and CXCR5 were downregulated on SF CD27^+ ^and CD27^- ^switch memory B cells compared with their circulating counterparts. SF CD27^+ ^and CD27^- ^switch memory B cells expressed at high levels the costimulatory molecule CD86 and the activation marker CD69.

**Conclusions:**

This study demonstrates for the first time an expansion of activated switch memory B cells and of IgG-secreting plasma blasts in the SF from oligoarticular JIA patients. Memory B cells belonged to either the CD27^+^or the CD27^- ^subsets and expressed CD86, suggesting their involvement in antigen presentation to T cells. Patterns of chemokines-receptor expression on CD27^+ ^and CD27^- ^switch memory B cells delineated potential mechanisms for their recruitment to the inflamed joints.

## Introduction

Juvenile idiopathic arthritis (JIA) is a heterogeneous condition classified into different subtypes according to the symptoms at onset [1]. Oligoarticular JIA is the most frequent form (26% to 56% of all JIA) and is characterized by early disease onset, asymmetric arthritis, high prevalence of iridocyclitis, peculiar HLA association (HLA-DRB1*1101, DRB1*0801, DPB1*0201), and the presence of antinuclear antibodies. In the majority of these patients, the disease remains confined to a limited number of joints (persistent oligoarticular JIA) and has a favorable outcome characterized by a high frequency of self-remission (as reviewed in [2]). Approximately one third of patients with oligoarticular onset experience progression toward a more aggressive form, characterized by the involvement of five or more joints after the first 6 months of disease (extended oligoarticular JIA). In 10% to 30% of JIA patients, the disease shows symmetric involvement of more than four joints, with an erosive course during the first 6 months of disease (polyarticular-onset JIA). A small proportion of these patients (3% to 5% of all JIA patients) display positivity for rheumatoid factor (RF) [2]. The systemic-onset JIA is observed in 4% to 17% of patients and is characterized by a severe systemic involvement (rash, fever, hepatosplenomegaly) associated with arthritis of variable severity that may evolve into an aggressive polyarticular course [2].

A distinctive feature of chronic inflammatory arthritides is the presence of synovial lymphocytic infiltrates that play a role in disease pathogenesis through the release of pro-inflammatory cytokines and other soluble mediators [3-5].

In adult rheumatoid arthritis (RA) and occasionally in JIA [6], these infiltrates may organize into follicle-like structures, according to a process known as "ectopic lymphoid neogenesis" [3-5]. Both T and B cells are detected in JIA infiltrates [6]. Whereas T cells are likely responding to autoantigens whose nature has been partially defined [7,8], the pathogenic role of B cells in JIA is less clear, because the vast majority of patients test negative for rheumatoid factors [2]. Nonetheless, synovial lymphocytic infiltrates have been recently correlated with the presence of serum anti-nuclear IgG antibodies in JIA patients [6]. The latter observation highlights the relevance of switch memory B cells in the production of these autoantibodies.

Furthermore, activated switch memory B cells can contribute to the pathogenesis of JIA by upregulating the expression of co-stimulatory molecules such as CD80 and CD86 and presenting antigens to T cells [9].

With this background, we here address the immunophenotypic and functional characterization of synovial B cells from JIA patients, with emphasis on switch memory B cells. The results obtained may be translationally relevant because RA patients can benefit from treatment with rituxan (Rituximab), a monoclonal antibody directed to the B cell-specific antigen CD20 [10,11], and preliminary evidence indicates that the same treatment may be efficacious in JIA patients [12].

## Materials and methods

### Patients

This investigation was approved by the Ethical Committee of the G. Gaslini Institute, Genoa, Italy. All biologic samples (blood, synovial fluid, or synovial tissue) from juvenile idiopathic arthritis (JIA) patients or healthy controls were obtained with informed consent of the patients' parents or the legal guardians. JIA individuals were classified according to ILAR Durban criteria [1]. Twenty-three of 25 patients had oligoarticular JIA, either persistent or extended, and two had RF-polyarticular JIA. All patients were in articular relapse at study. An intra-articular steroid injection in the previous 6 months was considered an exclusion criterion. The clinical characteristics and ongoing treatment are reported in Table 1. The two RF-polyarticular JIA cases were included in the extended JIA group for statistical purposes, because of the limited number of patients studied.

**Table 1:** Clinical and laboratory features of JIA patients enrolled in the study

Patient	JIA form	Disease duration (years)	Number of active joints	CRP (mg/dl)	ANA	Treatment
1	Oligo pers	8	1	1.32	Pos	NSAID
2	Oligo pers	6	1	<0.46	Pos	---
3	Oligo pers	2	2	0.83	Pos	---
4	Oligo pers	6	3	0.76	Pos	NSAID
5	Oligo pers	8	2	1.85	Pos	NSAID
6	Oligo pers	1	2	<0.46	Pos	NSAID
7	Oligo pers	5	1	<0.46	Pos	---
8	Oligo pers	10	1	<0.46	Neg	---
9	Oligo pers	6	1	0.61	Pos	---
10	Oligo pers	1	1	<0.46	Pos	---
11	Oligo pers	5	1	<0.46	Pos	---
12	Oligo pers	1	1	<0.46	Pos	NSAID
13	Oligo pers	9	1	<0.46	Pos	NSAID
14	Oligo ext	6	2	8.3	Pos	NSAID, MTX
15	Oligo ext	6	6	6.59	Pos	NSAID
16	Oligo ext	3	4	3.5	Pos	NSAID, MTX
17	Oligo ext	11	1	<0.46	Pos	MTX
18	Oligo ext	10	8	1.25	Pos	NSAID
19	Oligo ext	10	4	2.53	Pos	CyA, MTX
20	Oligo ext	1	4	0.87	Pos	NSAID
21	Oligo ext	2	5	1.34	Pos	NSAID
22	Oligo ext	10	1	0.6	Pos	MTX
23	Oligo ext	2	1	<0.46	Pos	NSAID, MTX
24	Poly RF	5	2	0.87	Pos	MTX
25	Poly RF	9	1	0.76	Neg	NSAID, adalimumab

Olig pers = oligoarticular persistent; oligo ext = oligoarticular extended; CRP = C-reactive protein (n.v. < 0.46 mg/dl); ANA = antinuclear antibody; pos = positive; neg = negative; NSAID = nonsteroidal anti-inflammatory drug = MTX = methotrexate; CyA = cyclosporine A.

### Cell isolation

Mononuclear cells (MNCs) were isolated with Ficoll-Hypaque density gradients (Sigma Chemical Company, St. Louis, MO) from synovial fluid (SF) and peripheral blood (PB) of 25 patients with JIA or 20 healthy individuals, comparable with respect to mean age (no clinical or laboratory evidence for inflammatory or infectious disorders in the 4 weeks before testing). MNCs were frozen in a solution containing 10% dimethyl sulfoxide (Sigma), and stored in liquid nitrogen until tested. Cells were cultured in RPMI 1640 medium supplemented with 10% fetal bovine serum (Sigma).

### Flow cytometry

The following monoclonal antibodies (mAbs) were used: CD19-phycoerythrin(PE)-cyanin(Cy)7, CD38-PerCP/Cy5, CD27-PerCP/Cy5 from Beckman Coulter (Marseille, France); CD3-allophycocyanin (APC)-Cy7, CD14-APC-Cy7, CD56-biotin and CD16-APC-Cy7, streptavidin-APC-Cy7, CD10-PE; CD24 fluorescein isothiocyanate (FITC), CD20-PE, CD27-PE, CD27-FITC, CD80-FITC; CD86-PE from BD Pharmingen (San Diego, CA, USA); CD10-FITC from Biolegend (San Diego, CA, USA); PE-conjugated anti-human IgD mAb from Dako (Glostrup, Denmark); anti-human immunoglobulin (Ig)G, IgA, and IgM-allophycocyanin (APC) from Jackson Immunoresearch Laboratories (West Grove, PA, USA); PE-conjugated anti-CC chemokine receptor CCR1-CCR9 mAbs from R&D Systems Inc. (Minneapolis, MN, USA); unconjugated anti-CXC chemokine receptor CXCR1, CXCR2, and CXCR3 mAbs from Serotec Inc. (Raleigh, NC, USA); and PE-conjugated anti-CXCR4 and CXCR5 mAbs from R&D Systems. Cell staining and flow-cytometric analysis were performed as reported [13] by using an FACSCanto (Becton-Dickinson). On average, 10^4 ^events were acquired and analyzed by using the CellQuest software.

The gates and the marker combinations used to analyze B-cell subpopulations are detailed later. In all flow-cytometry experiments performed in this study, the first step was the exclusion of non-B cells stained with a combination of the following antibodies and detected in a single channel: CD3, CD14, CD16, and CD56, all labeled with APC-Cy7.

Naïve B cells were detected as IgD^+^, IgM^+ ^cells after gating on CD19^+ ^cells. CD27^+ ^IgM memory B cells were detected as IgG^-^, IgA^- ^cells after gating on CD19^+ ^cells and subsequently on CD27^+ ^cells. CD27^+ ^switch memory B cells were detected as IgG^+^, IgA^+ ^cells after gating on CD19^+ ^cells and subsequently on CD27^+ ^cells. CD27^- ^switch memory B cells were detected as IgG^+^, IgA^+ ^cells after gating on CD19^+ ^cells and subsequently on CD27^- ^cells. Transitional B cells were detected as IgM^+^, IgD^+ ^cells after gating on CD19^+ ^cells and subsequently on CD24^high^, CD38^high ^cells. Germinal center-like B cells were detected as CD10^+ ^cells after gating on CD19^+ ^cells. Plasma blasts were detected as CD27^high^, CD20^+/- ^cells after gating set on CD19^+ ^cells.

### Immunohistochemistry

Synovial-membrane samples were obtained from three JIA patients undergoing synovectomy (two patients) or arthroscopic biopsy (one patient). Two independent tissue samples were collected from each patient, fixed in formalin 10%, and embedded independently in paraffin blocks or optimal cutting temperature (OCT) compound, and snap frozen in liquid nitrogen-cooled isopentane. Immunohistochemical labeling was performed with a three-step immunoperoxidase technique. Formalin-fixed, paraffin-embedded tissue sections were incubated at room temperature for 30 minutes with anti-CD20 mAb (clone L26, DakoCytomation), anti-CD3 polyclonal Ab (DakoCytomation), anti-CD138 mAb (clone MI15, DakoCytomation), anti-CD19 mAb (clone19C02, Neomarkers), anti-CD27 mAb (clone137B4, Neomarkers), anti-IgA mAb (clone 6E2C1, DakoCytomation), anti-IgG mAb (clone A57H, DakoCytomation), and anti-IgM mAb (clone R1/69, DakoCytomation). Sections were subsequently reacted at room temperature with anti-mouse Ig antibody conjugated to peroxidase-labeled dextran polymer (DakoCytomation). Chromogenic diaminobenzidine substrate was applied, and slides counterstained with Mayer's hematoxylin.

### ELISPOT assay

Cells secreting IgG, IgM, or IgA were detected with an ELISPOT assay, as reported [13], by using purified goat anti-human IgG or IgA or IgM from Southern Biotechnology Associates (Birmingham, AL, USA). After washing and blocking with PBS containing 1% BSA for 30 minutes, serial dilutions of cultured B cells were added and incubated overnight at 37°C. Before plating, cultured B cells were washed 5 times with complete medium to eliminate the Ig present in the supernatants. Plates were washed and incubated with isotype-specific secondary antibodies, followed with streptavidin-HRP (Sigma). The assay was developed with AEC (Sigma) as the chromogenic substrate. ELISPOT plates were analyzed blindly with the bioreader 3000 BIOSYS.

### Statistical analysis

Data were reported in terms of medians, minimum and maximum values, or first and third quartiles. The nonparametric analysis of variance (Kruskal-Wallis test) was used to compare quantitative parameters among three groups of observations (for example, to compare the number of naïve B cells in SF with respect to PB and to controls' PB); the Dunn test was used as an *a posteriori *test. The Mann-Whitney *U *test was used to compare quantitative variables between two groups of observations, and the Bonferroni correction was applied to avoid multiple comparisons error (P_B_) (for example, to compare number of CCR5^+^, CCR6^+^, CCR7^+^, CCR8^+^, and CCR9^+ ^cells in the SF versus the PB compartment). A *P *value less than 0.05 was considered statistically significant. Statistical analyses were performed by using Graph Pad Prism 3 software and the statistical package, Statistica 6 (StatSoft Inc., Tulsa, OK, USA).

## Results

### B-cell subset characterization in synovial fluid from oligoarticular JIA patients

We first performed a multicolor flow-cytometric analysis of MNC isolated from SF and paired PB samples of 25 JIA patients, as well as from PB samples of 20 age-matched controls. CD19^+ ^cells were less numerous in SF than in paired PB (SF percentage of positive cells: median, 0.9; range, 0.1 to 3.1; PB percentage of positive cells: median, 12.3; range, 4.6 to 22.0, *P *< 0.0001; SF absolute number: median, 24.7; range, 3.0 to 65.3; and PB absolute number: median, 297.3; range, 34.8 to 1,067; *P *< 0.0001). CD19^+ ^cells in control PB (percentage of positive cells: median, 11.0; range, 4.8 to 28.9; absolute number median, 283.6; range, 123.7 to 985.1) did not differ from those in patient PB (see values in parentheses in the previous sentence).

**Figure 1:**
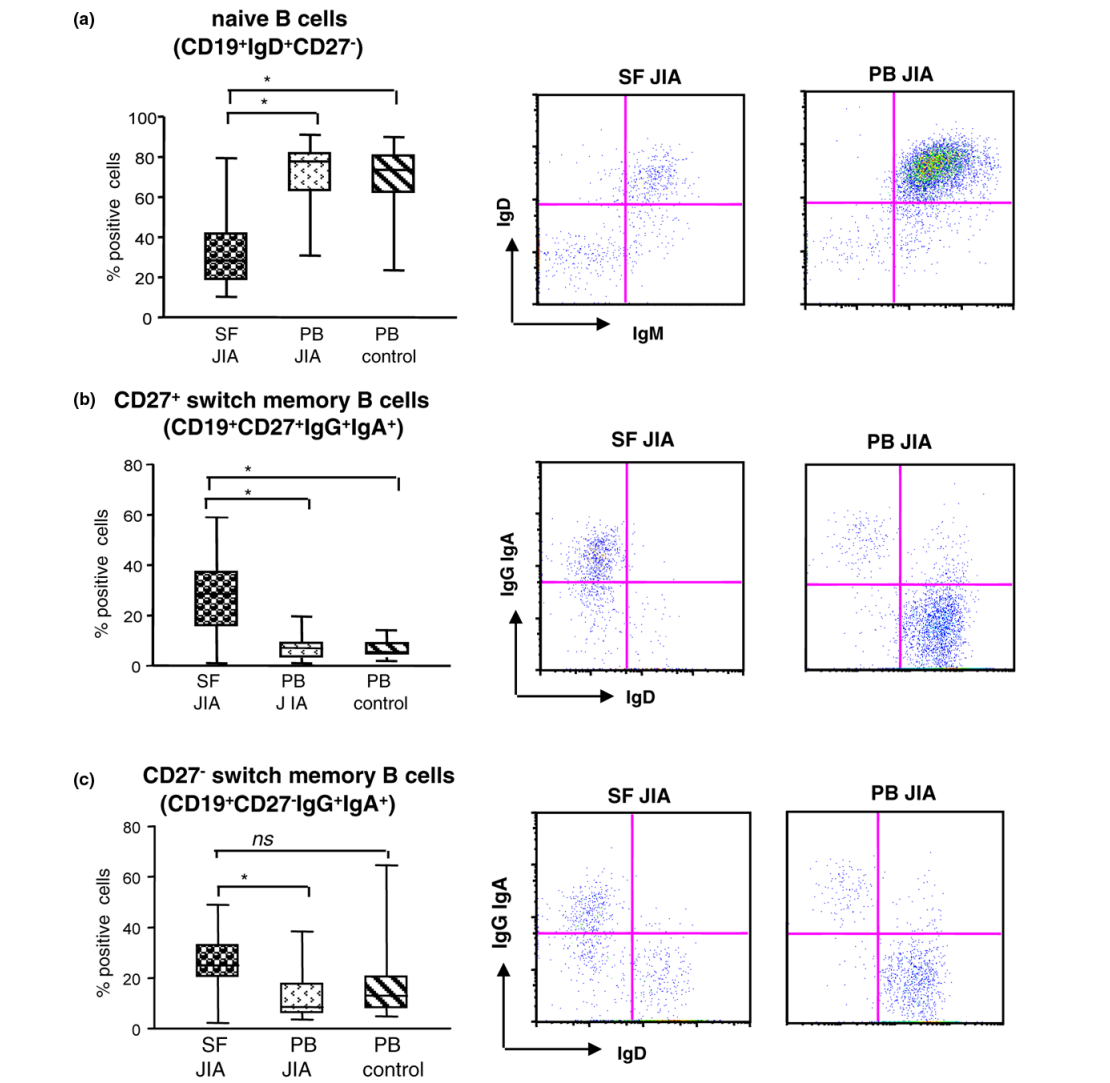
Flow cytometry of B-cell subsets in synovial fluid (SF) and peripheral blood (PB) from juvenile idiopathic arthritis (JIA) patients.  Mononuclear cells (MNCs) from SF and PB of 25 JIA patients, as well as from PB of 20 age-matched controls, were stained with different B cell-specific antibodies and analyzed with flow cytometry. The first gate was set on CD3^-^, CD14^-^, CD16^-^, and CD56^- ^cells (non-B cell lineage cells) followed by gating on CD19^+ ^cells. Results are expressed in a box plot (left panel) as median percentage of positive cells, minimum and maximum value. * *P *< 0.01. One representative dot-blot for SF (middle panel) and PB (right panel) is shown.

Naïve (CD19^+^IgD^+^CD27^-^) B cells [14] were less abundant in SF than in patient and control PB (Dunn test; *P *< 0.01), whereas they were equally represented in control and JIA PB (Figure 1A). B cells with a germinal center-like phenotype **(**CD19^+^, CD10^+ ^cells) [15] were virtually absent from both SF andpatient or control PB (data not shown).

The percentage of CD19^+^CD27^+ ^memory B cells was higher in SF than in patient PB (SF median, 44.2; range, 10.0 to 73.0; *n* = 25; PB median, 16.4; range, 4.1 to 34.9; *n* = 25; *P *< 0.0001). No difference was observed between memory B cells from JIA or control PB (median, 10.5; range, 4.7 to 37.0; *n* = 16).

Two subsets of CD19^+^CD27^+ ^memory B cells have been identified: (a) IgM memory (CD19^+^CD27^+^IgG^-^IgA^-^), and (b) class switch memory (CD19^+^CD27^+^IgG^+^IgA^+^) B cells [14,16]. The percentage of IgM memory B cells in SF did not differ from that in patient PB, which showed values comparable to those in control PB (SF median, 11.0; range, 0.0 to 21.0; *n* = 25; paired PB median, 8.0; range, 2.4 to 27.6; *n* = 25; control PB median, 8.2; range, 4.8 to 17.9; *n* = 16).

CD27^+ ^switch memory B cells were enriched in SF versus patient PB (Dunn test; *P *< 0.01) (Figure 1B). The same cell subset was similarly represented in control and patient PB (Figure 1B).

Recently, a novel subset of switch memory B cells lacking CD27 expression (CD19^+^CD27^-^IgG^+^IgA^+^) was identified [17-19]. CD27^-^switch memory B cells were more abundant in SF than in patient PB (Dunn test; *P *< 0.01), whereas they were present in similar proportions in control and patient PB (Figure 1C).

CD19^+^CD27^-^IgG^+^IgA^+ ^switch memory B cells can be further subdivided into two subsets according to the expression of the FcHR4 surface marker [17-19]. SF CD19^+^CD27^-^IgG^+^IgA^+ ^switch memory B cells tested negative for FcHR4 expression.

**Figure 2:**
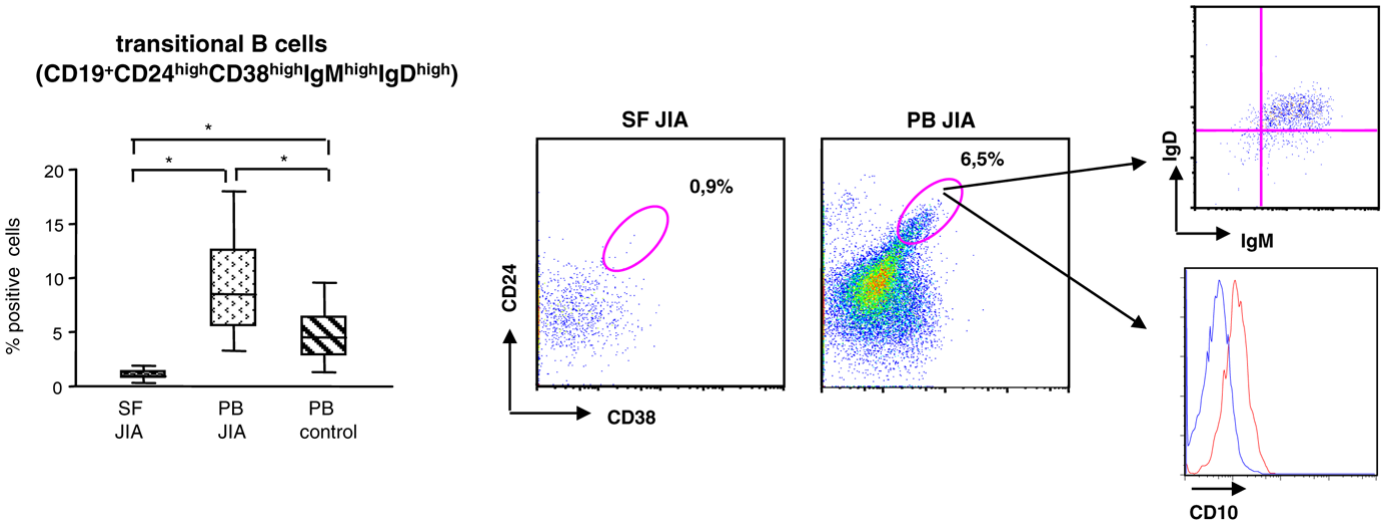
Transitional B cells in synovial fluid (SF) and peripheral blood (PB) in juvenile idiopathic arthritis (JIA) patients.  Cells from SF and PB of JIA patients, as well as from control PB, were analyzed with flow cytometry by gating on CD3^-^, CD14^-^, CD16^-^, and CD56^- ^cells (non-B cell lineage cells) followed by gating on CD19^+ ^cells and then on CD24^high^, CD38^high ^cells. Results are expressed in a box plot (left panel) as median percentage of positive cells, minimum and maximum value. *P < 0.01 (patient PB versus SF) and *P *= 0.05 (patient versus control PB). One representative dot-blot for SF (middle panel) and PB (right panel) is shown. Insets on the right side of the Figure show that gated CD24^high^and CD38^high ^B cells express immunoglobulin M and immunoglobulin D (upper inset) and CD10 (lower inset).

CD19^+^CD24^high^CD38^high^IgM^high^IgD^high ^transitional B cells [20] were virtually absent in SF compared with patient and control PB (Dunn test; *P *< 0.01) (Figure 2) and were more abundant in patient than in control PB (Dunn test; *P *= 0.05) (Figure 2). It has been reported that transitional B cells can express CD10 [21]. To address this issue, we stained transitional B cells from patient PB for CD10 and found that the latter marker was expressed by these cells (15% to 20%; range from four experiments with different samples) (Figure 2). Patients with oligoarticular persistent JIA who were receiving NSAID treatment or were untreated at study did not show significant differences in the percentages of all the mentioned B-cell subsets (naïve B cells, CD27^+ ^and CD27^- ^switch memory B cells, and plasma blasts), either in SF or in PB. Similar results were obtained from the comparison of patients with persistent or extended oligoarticular JIA (not shown).

**Figure 3:**
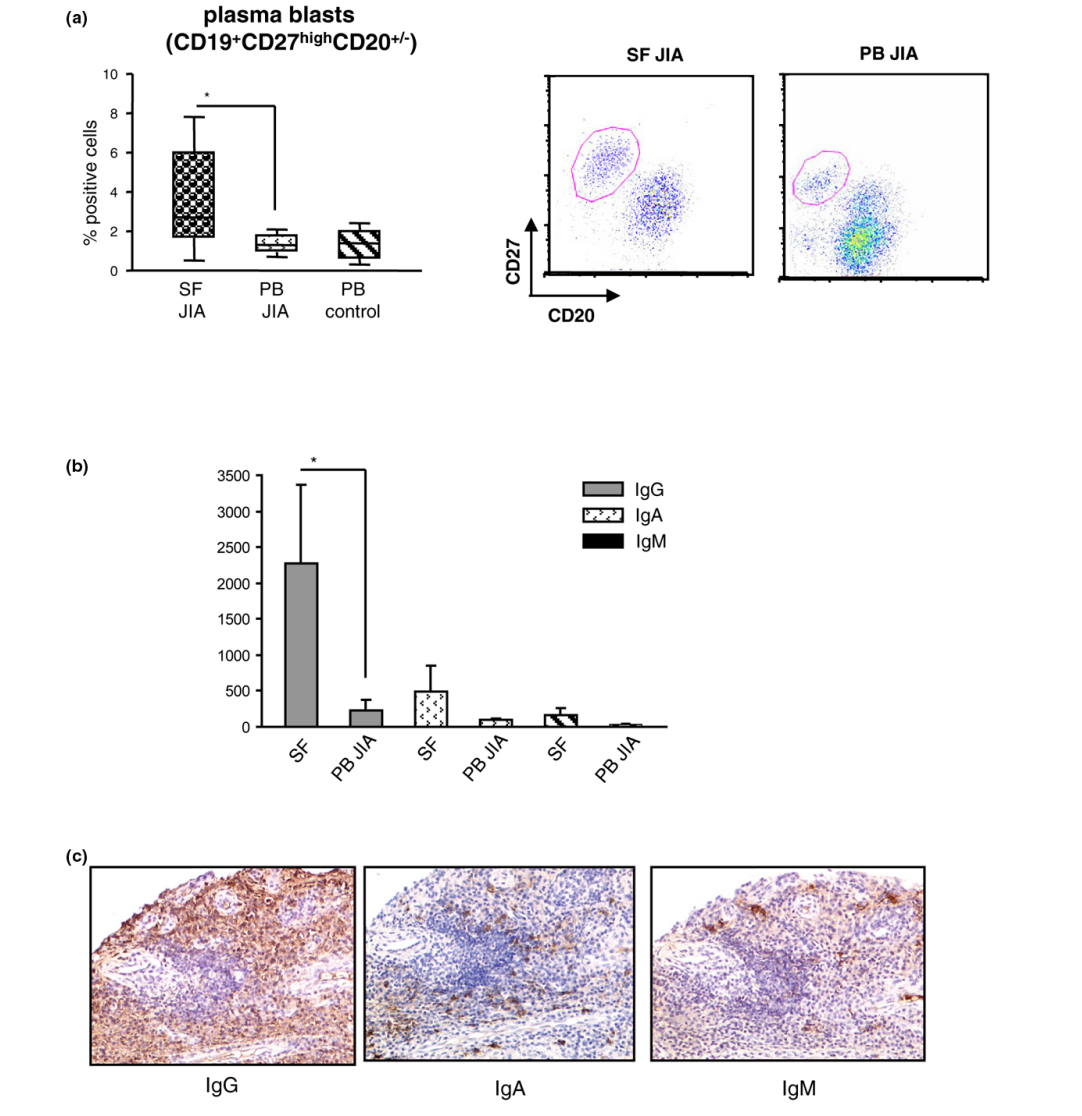
Plasma blasts and Ig-secreting cells in synovial fluid (SF), peripheral blood (PB), and synovial tissue from juvenile idiopathic arthritis (JIA) patients.  **(a) **Cells from JIA SF and PB, as well as from control PB, were analyzed with flow-cytometry gating, first on CD3^-^, CD14^-^, CD16^-^, and CD56^- ^cells (non-B cell lineage cells), and then on CD19^+^cells, and finally analyzed for CD27 and CD20 expression. Results are expressed in a box plot as median percentage of positive cells, minimum and maximum value. *P < 0.05. One representative dot-blot for SF (middle panel) and PB (right panel) is shown. **(b) **IgG, IgA, or IgM CD19^+^immunoglobulin-secreting cells (ISCs) were detected in SF and PB from four JIA patients with ELISPOT. Results are expressed as mean ISC ± SD. *P = 0.028. **(c) **Serial synovial tissue sections from three JIA patients were stained with anti-IgG, anti-IgA, or anti-IgM mAbs by using the peroxidase method (brown staining).

CD19^+^CD27^high^CD20^+/- ^plasma blasts [22] were increased in SF versus patient PB (Dunn test; *P *< 0.05) (Figure 3A). The percentage of plasma blasts in control PB and JIA PB was similar (Figure 3A). The percentage of plasma blasts was higher in SF from extended than from persistent oligoarticular JIA patients (*P *= 0.014).

ELISPOT experiments showed higher numbers of CD19^+ ^IgG-secreting cells in SF versus patient PB (*P *= 0.028) (Figure 3B). CD19^+ ^IgA- and IgM-secreting cells were equally represented in patient SF and PB (Figure 3B). Similar percentages of IgG-, IgM-, or IgA-secreting B cells were found in patient and control PB (not shown).

Immunohistochemical staining for IgG, IgA, and IgM in synovial tissue sections showed a predominance of IgG-secreting cells. Lower proportions of IgA- and IgM-secreting cells were also detected (Figure 3C).

Chemokine receptor and costimulatory molecule expression in CD27+ and CD27- switch memory B cells from synovial fluid of oligoarticular JIA patients Next, CD27^+ ^and CD27^- ^switch memory B cells were characterized with flow cytometry for the expression of CC chemokine receptors (R) (CCR1-CCR9) and CXCR (CXCR1-CXCR5), as well as for the CD80 and CD86 costimulatory molecules.

#### CD27^+ ^switch memory B cells

**Figure 4:**
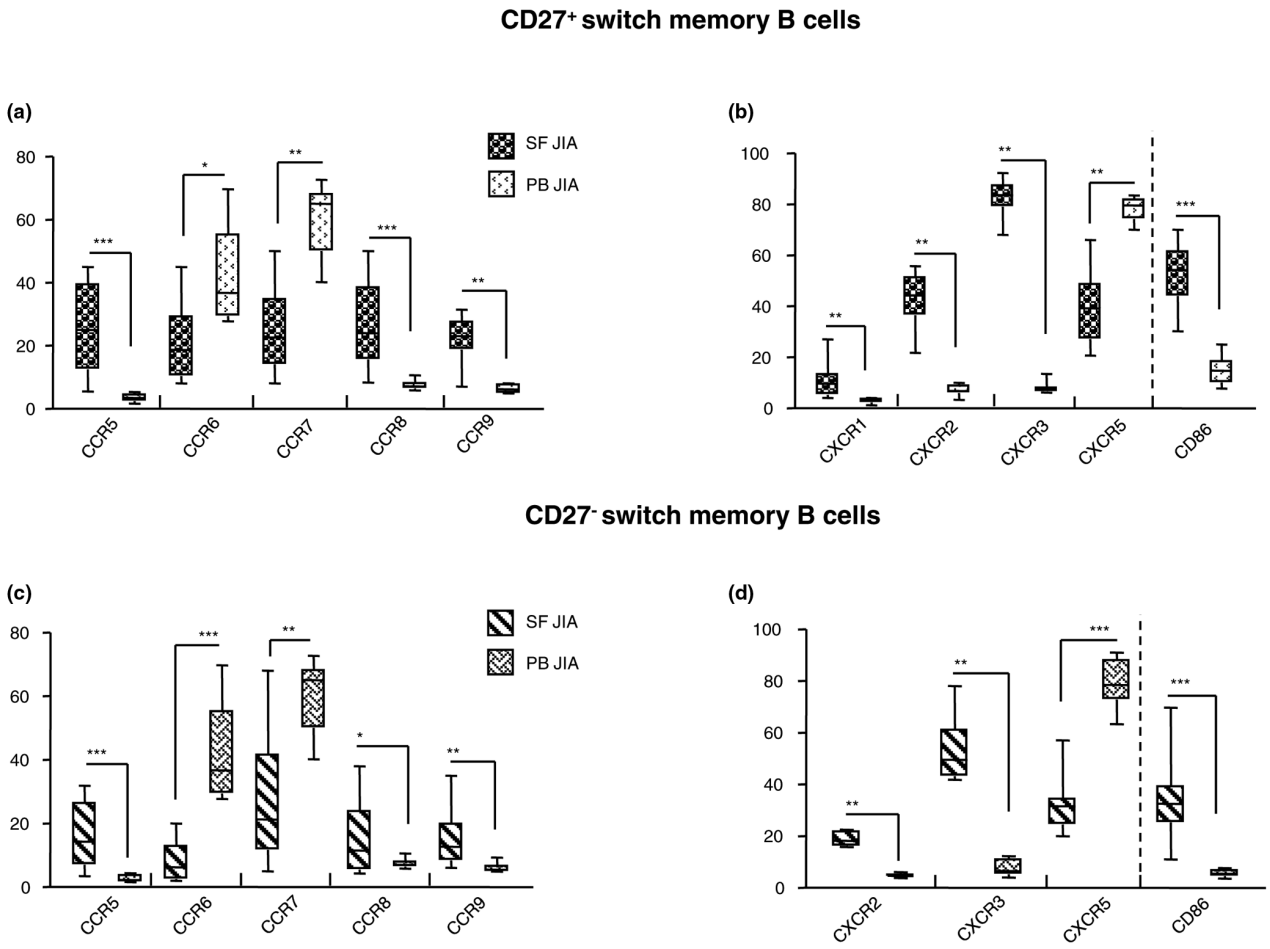
Chemokine receptor and CD86 expression on switch memory B cells from juvenile idiopathic arthritis (JIA) patients.  Cells from JIA synovial fluid (SF) and peripheral blood (PB) were analyzed with flow-cytometry gating first on CD3^-^, CD14^-^, CD16^-^, and CD56^- ^cells (non-B cell lineage cells), then on CD19^+^cells, and subsequently on CD27^+ ^or CD27^- ^cells before being analyzed for individual chemokines-receptor expression. **(a) **CC chemokine receptor expression on SF and PB CD27^+^switch memory B cells. Results are expressed in a box plot as median percentage of positive cells, minimum and maximum value. *** *P *= 0.0002; *P *= 0.0003. **P = 0.001; *P *= 0.004. *P = 0.019. **(b) **CXC chemokine receptor and CD86 expression on SF and PB CD27^+^switch memory B cells. Results are expressed in a box plot as median percentage of positive cells, minimum and maximum value. ***P = 0.0001. **P = 0.001; *P *= 0.002. **(c) **CCR expression on SF and paired PB CD27^- ^switch memory B cells. Results are expressed in a box plot as median percentage of positive cells, minimum and maximum value. ***P = 0.0001; *P *= 0.0003. **P = 0.0034; *P *= 0.0041. **(d) **CXCR and CD86 expression on SF and paired PB CD27^-^switch memory B cells. Results are expressed in a box plot as median percentage of positive cells, minimum and maximum value. ***P = 0.0001; *P *= 0.0006. **P = 0.0011.

CCR5^+^, CCR8^+^, and CCR9^+ ^cells were significantly increased, whereas CCR6^+ ^and CCR7^+ ^cells were decreased in SF versus patient PB (Figure 4A and Table 2). CXCR1^+^, CXCR2^+^, and CXCR3^+ ^cells were significantly more abundant, whereas CXCR5^+ ^cells were less numerous in SF than in patient PB cells (Figure 4B and Table 2). No difference in CXCR4 expression was observed between SF and patient PB cells.

**Table 2:** Chemokine-receptor expression in synovial fluid and peripheral blood CD27^+ ^switch memory B cells

Chemokine receptors	Synovial fluid^a^(n = 13)	Peripheral blood^a^(n = 13)	*P *value^b^
CCR1	7.0 (2.0-18)	6.5 (3.3-8.5)	ns
CCR2	6.7 (2.4-28)	5.8 (4.4-6.9)	ns
CCR3	6.9 (2.0-27.3)	6.5 (5.2-7.7)	ns
CCR4	6.6 (2.3-21.5)	5.3 (2.9-6.4)	ns
CCR5	25.0 (5.5-45)	36.8 (27.8-69.7)	0.0003
CCR6	18.7 (8.0-45)	3.4 (1.6-5.3)	0.019
CCR7	22.6 (8.0-50)	65.1 (40.2-72.7)	0.001
CCR8	24.0 (8.3-50)	7.1 (5.8-10.6)	0.0002
CCR9	23.1 (6.1-76)	6.2 (4.9-8)	0.004
CXCR1	9.7 (4.0-27.0)	3.0 (1.2-4.1)	0.001
CXCR2	44.4 (21.8-55.7)	6.8 (3.4-10.0)	0.002
CXCR3	83.6 (68.0-92.3)	7.3 (6.2-13.5)	0.002
CXCR4	49.0 (37.3-63.6)	72.2 (35.8-85.3)	ns
CXCR5	39.3 (20.7-66.0)	79.5 (70.0-83.5)	0.001

^a^Results represent median percentage of positive cells, as assessed with flow cytometry. Values in parentheses are minimum and maximum values. ^b^*P *values were calculated by comparing the results of JIA SF and PB cell staining. ns = not significant.

Finally, CCR and CXCR expression was similar in patient and control PB cells (not shown). SF cells expressed higher levels of the CD86 costimulatory molecule than did patient PB cells (*P *= 0.0001) (Figure 4B). In contrast, CD80 expression was similar (SF CD80 median, 7.5; range, 2.0 to 21.9; n = 15; PB CD80 median, 15.2; range, 13.1 to 21.4; n = 10). Likewise, CD86 and CD80 expression on cells from patient and control PB (not shown) was comparable.

To achieve insight into the activation state of CD27^+ ^switch memory B cells, the latter cells from SF and PB were stained with mAbs to CD25, CD69, and HLA-DR. Percentages of CD69^+ ^and CD25^+ ^cells from SF were significantly higher than those in PB (SF CD69 median, 12.9; range, 8.8 to 17.0; n = 5; PB CD69 median, 2.5; range, 0.4 to 4.5; n = 5; *P *= 0.007; SF CD25 median, 1.5; range, 0.9 to 1.8; n = 5; PB CD25 median, 0.4; range, 0.2 to 0.5; n = 5; *P *= 0.007), whereas the expression of HLA-DR was similar in the two compartments (SF HLA-DR median, 90.9; range, 74.2 to 92.9; n = 5; PB HLA-DR median, 81.8; range, 71.4 to 98; n = 5).

#### CD27^- ^switch memory B cells

CCR5^+^, CCR8^+^, and CCR9^+ ^cells were significantly increased, whereas CCR6^+ ^and CCR7^+ ^cells were decreased in SF versus patient PB (Figure 4C and Table 3). CXCR2^+ ^and CXCR3^+ ^cells were significantly more numerous, whereas CXCR5^+ ^cells were less abundant in SF than in patient PB cells (Figure 4D and Table 3). CXCR4 expression was similar in SF and patient PB cells (not shown). Few CXCR1^+ ^cells were detected in SF and patient PB (not shown). Expression of all CCR and CXCR was similar in control (data not shown) and patient PB cells.

**Table 3:** Chemokine-receptor expression in SF and PB CD27^- ^switch memory B cells

Chemokine receptors	Synovial fluid^a^(n = 13)	Peripheral blood^a^(n = 13)	*P *value^b^
CCR1	5.4 (2.0-30)	5.1 (4.7-5.9)	ns
CCR2	5.7 (1.0-34)	5.2 (3.4-6.6)	Ns
CCR3	7.1 (4.0-28.1)	6.5 (5.7-7.0)	ns
CCR4	6.0 (2.3-19.7)	5.1 (3.9-5.9)	ns
CCR5	16.1 (3.4-31.9)	2.1 (1.6-4.4)	0.0001
CCR6	6.3 (2.0-20)	36.8 (27.8-69.7)	0.0003
CCR7	21.3 (5.0-68)	65.1 (40.2-72.7)	0.0034
CCR8	11.5 (4.3-38)	4.9 (3.0-7.0)	0.025
CCR9	12.7 (6.1-35)	5.5 (4.9-9.3)	0.0041
CXCR1	4.2 (0.2-9.1)	1.5 (1-3.5)	ns
CXCR2	18.2 (15.8-22.5)	4.7 (3.8-6.1)	0.0011
CXCR3	49.5 (41.8-78)	6.5 (4-12.3)	0.0011
CXCR4	43.1 (30.5-54.8)	67.5 (33.5-90.8)	ns
CXCR5	31.6 (20-57.1)	78.4 (63.4-91)	0.0006

^a^Results represent median percentage of positive cells as assessed with flow cytometry. Values in parentheses are minimum and maximum values. ^b^*P *values were calculated by comparing the results of JIA SF and PB cell staining. ns = not significant.

Finally, SF cells expressed significantly higher levels of CD86 than did patient PB cells (*P *= 0.0001) (Figure 4D). In contrast, CD80 expression was similar in SF and patient PB (SF CD80 median, 3.3; range, 1.2 to 10.3; *n* = 15; PB CD80 median, 4.9; range, 3.8 to 6.1; *n* = 10). CD86 and CD80 expression in patient and control PB (not shown) was comparable. The percentage of CD69^+ ^cells was significantly higher in SF than in PB (SF CD69 median, 11.3; range, 4.0 to 14.2; *n* = 5; PB CD69 median, 0.4; range, 0.1 to 1.2; *n* = 5; *P *= 0.007). In contrast, CD25^+ ^and HLA-DR^+ ^cells were significantly more abundant in SF than in PB (SF CD25 median, 1.1; range, 0.6 to 2.1; *n* = 5; PB CD25 median, 0.1; range, 0.03 to 2.0; *n *= 5; SF DR median, 83.8; range, 58.7 to 96.9; *n* = 5; PB DR median, 91.2; range, 82.9 to 97.1; *n* = 5).

### Characterization of B-cell infiltrates in synovial tissue from JIA patients

**Figure 5:**
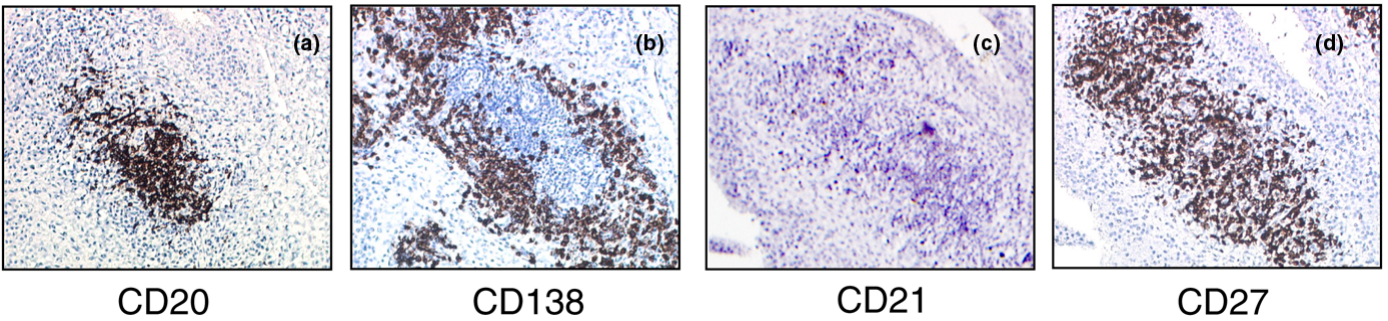
Histologic analysis of synovial tissue sections from juvenile idiopathic arthritis (JIA) patients.   Serial synovial tissue sections from three JIA patients were stained with CD20 **(a)**, CD138 **(b)**, CD21 **(c)**, and CD27 **(d) **mAbs by using the peroxidase method. CD20^+ ^B cells cluster within lymphoid aggregates, whereas CD138^+ ^plasma cells localize at the periphery of such aggregates. Staining for CD21, a follicular dendritic cell marker, is negative, consistent with the absence of follicular organization. CD27^+ ^cells are found both inside and outside the lymphoid aggregates.

In three JIA cases (one with oligoarticular persistent and two with oligoarticular extended JIA), histologic analysis of synovial tissue sections demonstrated the presence of lymphoid aggregates with predominant perivascular distribution [6]. Clusters of CD20^+ ^B cells (Figure 5A) and CD3^+ ^T cells (not shown) were detected within lymphoid aggregates, whereas CD138^+ ^plasma cells were found at the periphery of such aggregates (Figure 5A and 5B). Consistent with a previous study [6], these aggregates were not organized in follicle-like structures, because they tested negative for the expression of the follicular dendritic cell marker CD21 (Figure 5C).

CD27^+ ^cells were detected both within and around lymphoid aggregates, consistent with CD27 expression by memory B cells and plasma blasts/plasma cells [23], as well as by memory T cells [24] (Figure 5D).

## Discussion

In this study, CD27^+ ^and CD27^- ^switch memory B cells from oligoarticular JIA patients were found to be significantly enriched in SF compared with PB, whereas CD27^+^IgM^+ ^B cells were equally represented in the two compartments.

A previous study showed that CD27^- ^switch memory B cells from normal subjects and systemic lupus erythematosus (SLE) patients had substantial levels of Ig mutations but lower than conventional CD27^+ ^switch memory B cells [18]. The former B cells expanded in the peripheral blood from SLE patients, and this expansion correlated with high disease activity and high titers of disease-specific autoantibodies [18]. In contrast, the frequency of CD27^- ^switch memory B cells was reported to be normal in the peripheral blood from adult RA patients [18]. Accordingly, in our study, these cells were detected in similar proportions in the peripheral blood of oligoarticular JIA patients and controls.

CD27^- ^switch memory B cells from both PB and SF of our patients did not express the FcRH4 surface marker, as reported in SLE patients [18]. FcRH4^- ^CD27^- ^switch memory B cells may be more responsive to activation and may expand more easily in autoimmune diseases than their CD27^-^, FcRH4^+ ^counterparts, because FcRH4 is a potent inhibitor of B-cell signaling [25].

All patients tested had an articular disease relapse. No significant differences in the proportions of most SF or PB B-cell subpopulations were detected in patients subdivided according to ongoing treatment or disease subtype (*i.e*., persistent versus extended oligoarticular JIA). The only exception was represented by plasma blasts that were significantly more numerous in SF from extended than from persistent oligoarticular JIA patients.

Memory B cells migrate selectively to inflamed tissues [26,27]. To gain more insight into the mechanisms of CD27^+ ^and CD27^- ^switch memory B cell recruitment to the inflamed joints, we investigated their expression of a panel of chemokine receptors. CCR5, CCR8, CCR9, CXCR2, and CXCR3 were upregulated, whereas CCR6, CCR7, and CXCR5 were downregulated on both SF memory B-cell subsets versus their PB counterparts. In contrast, CXCR1 was found to be upregulated in SF CD27^+ ^but not CD27^- ^switch memory B cells, although the mechanisms underlying such a difference are unknown.

These findings suggest that CD27^+ ^and CD27^-- ^switch memory B cells from oligoarticular JIA patients share a common set of chemokine receptors, likely mediating their attraction to the affected joints, where the respective chemokine ligands [28] are abundantly produced as a consequence of chronic inflammation [29-31]. Unfortunately, the chemotactic functionality of the chemokine receptors upregulated in switch memory B cells could not be investigated because of the paucity of the latter cells in SF.

In adulthood, transitional B cells generated in the bone marrow are released into the bloodstream and transported to the spleen, where they develop into long-lived mature B cells [20,32]. Here, we demonstrated that transitional B cells were virtually absent from the synovial fluid of JIA patients, whereas they were detected in paired blood samples and found to be significantly increased in comparison with control blood. Similar observations have been reported in patients with Sjögren syndrome and SLE [33,34].

In principle, the increased frequency of transitional B cells in peripheral blood from JIA patients as compared with that in normal controls might result from dysfunctional bone marrow production or altered recirculation. Studies performed in SLE and Sjögren disease would favor the latter hypothesis [33,34]. Expansion of transitional B cells in blood from HIV-infected and idiopathic CD4^+ ^T lymphocytopenia patients has been correlated with increased serum levels of interleukin-7 [35,36]. Whether a similar correlation applies also to our patients remains to be established.

In this study, CD138^+ ^plasma cells were detected in synovial tissue at the periphery of lymphoid aggregates, lacking follicular organization and containing CD20^+^, CD27^+ ^memory B cells admixed with T cells. Synovial tissue from oligoarticular JIA patients with active, long-standing disease was previously shown to be infiltrated with high numbers of plasma cells [37]. Accordingly, we found an enrichment for IgG-secreting plasma blasts in SF and a large amount of IgG-producing B cells in synovial tissue. The functional significance of these findings remains to be established.

We finally showed that SF CD27^+ ^and CD27^- ^switch memory B cells were activated, as assessed by the expression of CD69, and expressed high levels of the CD86, but not CD80, costimulatory molecule. Resting B cells are poor antigen-presenting cells because of the low surface expression of costimulatory molecules. Upregulation of CD86 on synovial memory B cells suggests that these cells can efficiently present antigen to and activate T cells, contributing to the persistence of chronic inflammation [38-40]. Increased frequency of B cells expressing costimulatory molecules also was reported in other human autoimmune diseases [41-44]. The dual role of synovial B cells as antibody-producing and antigen-presenting cells supports their important pathogenic role in JIA and provides a rationale for a clinical trial with the anti-CD20 mAb rituximab [12], which has already shown therapeutic efficacy in adult RA patients [10,11].

## Conclusions

This study demonstrates for the first time an expansion of switch memory B cells and IgG-secreting plasma blasts in the SF from oligoarticular JIA patients. These memory B cells belonged to both the CD27^+ ^and the CD27^- ^subsets and expressed CD86, suggesting their involvement in antigen presentation to T cells. Analysis of chemokines-receptor expression on CD27^+ ^and CD27^- ^switch memory B cells delineated potential mechanisms for their recruitment to the inflamed joints. The dual role of synovial B cells as antibody-producing and antigen-presenting cells supports their pathogenic role in JIA and provides a rationale for a clinical trial with the anti-CD20 mAb rituximab.

## Abbreviations

CCR: CC chemokine receptor; CXCR: CXC chemokine receptor; FITC: fluorescein isothiocyanate; JIA: juvenile idiopathic arthritis; mAb: monoclonal antibody; MNC: mononuclear cell; PB: peripheral blood; PE: phycoerythrin; RA: rheumatoid arthritis; SF: synovial fluid; SLE: systemic lupus erythematosus; ST: synovial tissue.

## Competing interests

The authors declare that they have no competing interests.

## Authors' contributions

ET and VP contributed equally to this work. AC designed and supervised *in vitro *studies, analyzed data, and wrote the paper. FF performed *in vitro *experiments (cell separation, flow-cytometric analysis). MG participated in the design of the study. AG performed histologic studies. AP performed statistical analysis. RG provided normal peripheral blood samples. CG supervised histologic studies. AM helped to draft the manuscript. ET contributed to research design, reviewed data, and wrote the paper. VP designed research, reviewed data, and wrote the paper. All authors read and approved the final manuscript.
